# A new locus for autosomal recessive congenital cataract identified in a Pakistani family

**Published:** 2010-02-16

**Authors:** Haiba Kaul, S. Amer Riazuddin, Afshan Yasmeen, Sadia Mohsin, Mohsin Khan, Idrees A. Nasir, Shaheen N. Khan, Tayyab Husnain, Javed Akram, J. Fielding Hejtmancik, Sheikh Riazuddin

**Affiliations:** 1 National Centre of Excellence in Molecular Biology, University of the Punjab, Lahore, Pakistan; 2 Allama Iqbal Medical College, University of Health Sciences, Lahore, Pakistan; 3 Ophthalmic Genetics and Visual Function Branch, National Eye Institute, National Institutes of Health, Bethesda, MD

## Abstract

**Purpose:**

To identify the disease locus for autosomal recessive congenital cataract in a consanguineous Pakistani family.

**Methods:**

All affected individuals underwent detailed ophthalmologic and medical examination. Blood samples were collected and DNA was extracted. A genome-wide scan was performed with polymorphic microsatellite markers on genomic DNA from affected and unaffected family members, and logarithm of odds (LOD) scores were calculated.

**Results:**

The clinical records and ophthalmological examinations suggested that all affected individuals have nuclear cataracts. Maximum LOD scores of 5.01, 4.38, and 4.17 at θ=0 were obtained with markers D7630, D7S657, and D7S515, respectively. Fine mapping refined the critical interval and suggested that markers in a 27.78 cM (27.96 Mb) interval are flanked by markers D7S660 and D7S799, which co-segregate with the disease phenotype in family PKCC108.

**Conclusions:**

We have identified a new locus for autosomal recessive congenital cataract, localized to chromosome 7q21.11-q31.1 in a consanguineous Pakistani family.

## Introduction

Congenital cataracts are one of the major causes of vision loss in children worldwide and are responsible for about one-third of cases of blindness in infants [1,2]. They can occur in an isolated fashion or as one component of a syndrome affecting multiple tissues. Nonsyndromic congenital cataracts have an estimated frequency of 1–6 per 10,000 live births [3]. They can lead to permanent blindness by interfering with the sharp focus of light on the retina, especially during the early developmental periods. Morphologically, different types of cataract are classified according to the part of the opacified lens, including nuclear, cortical, lamellar, sutural, polar, or subcapsular cataract [4].

Approximately one-third of congenital cataract cases are familial [5]. To date, over 23 independent autosomal dominant cataract loci have been reported. Conversely, fewer autosomal recessive cataract loci have been mapped. To date, 12 loci residing on chromosomes 1p34.3-p32.2, 1q21.1, 3p22–24.2, 6p23–24, 9q13–22, 16q21–22, 19q13, 19q13.4, 20p12.1, 21q22.3, 22q11, and 22q12.1 have been mapped, with six of these also causing autosomal dominant cataracts [6-17]. Of these loci, mutations in eight genes *GJA8*, *GCNT2*, *HSF4*, *LIM2*, *BFSP1*, *CRYAA, CRYβB1*, and *CRYβB3* have been identified [7,9,11,13-17].

Here, we report a new locus for autosomal recessive nuclear cataract in a large consanguineous Pakistani family (PKCC108). Genome-wide linkage analyses localized the critical interval to chromosome 7q, whereas fine mapping refined the critical interval to a 27.78 cM (27.96 Mb) flanked by markers D7S660 and D7S799 that co-segregates with the disease phenotype in PKCC108.

## Methods

### Clinical ascertainment

A total of 100 consanguineous Pakistani families with nonsyndromic cataract were recruited to participate in a collaborative study between the Center of Excellence in Molecular Biology, Lahore, Pakistan, and the National Eye Institute, Bethesda, MD, to identify new disease loci causing inherited visual diseases. Institutional Review Board (IRB) approval was obtained from the National Eye Institute and the National Centre of Excellence in Molecular Biology. The participating subjects gave informed consent consistent with the tenets of the Declaration of Helsinki. A detailed medical history was obtained by interviewing family members. Ophthalmic examinations were conducted with slit-lamp microscopy. Approximately 10 ml of blood samples were drawn from affected and unaffected members of the family and stored in 50 ml Sterilin® falcon tubes containing 400 μl of 0.5 M EDTA. Blood samples were kept at -20 °C for long-term storage. 

### DNA extraction

DNA was extracted by a nonorganic method, as described by Grimberg et al. with minor modifications [18]. Briefly, aliquots of 10 ml blood samples were mixed with 35 ml of TE buffer (10 mM Tris-HCl, 2 mM EDTA, pH 8.0) and the TE-blood mixture was centrifuged at 3,000 rpm for 20 min. The supernatant was discarded and the pellet was re-suspended in 35 ml of TE buffer and centrifuged at 3,000 rpm for 20 min. The TE washing was repeated for 2-3 times and the washed pellet was re-suspended in 2 ml of TE. 6.25 ml of protein digestion cocktail (50 μl [10 mg/ml] of proteinase K, 6 ml TNE buffer [10 mM Tris HCl, 2 mM EDTA, 400 mM NaCl] and 200 μl of 10% sodium dodecyl sulfate) was added to the re-suspended pellets and incubated overnight in a shaker (250 rpm) at 37 °C. The digested proteins were precipitated by adding 1 ml of 5 M NaCl, followed by vigorous shaking and chilling on ice for 15 min. The precipitated proteins were pelleted by centrifugation at 3,000 rpm for 20 min and removed. The supernatant was mixed with equal volumes of phenol/chloroform/isoamyl alcohol (25:24:1) and the aqueous layer containing the genomic DNA was carefully collected. The DNA was precipitated with isopropanol and pelleted by centrifugation at 4,000 rpm for 15 min. The DNA pellets were washed with 70% ethanol and dissolved in TE buffer. The DNA concentration was determined with a SmartSpec plus Bio-Rad Spectrophotometer (Bio-Rad, Hercules, CA).

### Genotype analysis

A genome-wide scan was performed with 382 highly polymorphic fluorescent markers from the ABI PRISM Linkage Mapping Set MD-10 (Applied Biosystems, Foster City, CA) having an average spacing of 10 cM. Multiplex polymerase chain reaction (PCR) was completed in a GeneAmp PCR System 9700 thermocycler (Applied Biosystems). Briefly, each reaction was carried out in a 5 μl mixture containing 40 ng genomic DNA, various combinations of 10 mM dye-labeled primer pairs, 0.5 ml 10× GeneAmp PCR Buffer (Applied Biosystems), 1 mM dNTP mix, 2.5 mM MgCl_2_, and 0.2 U Taq DNA polymerase (Applied Biosystems). Initial denaturation was performed for 5 min at 95 °C, followed by 10 cycles of 15 s at 94 °C, 15 s at 55 °C, and 30 s at 72 °C and then 20 cycles of 15 s at 89 °C, 15 s at 55 °C, and 30 s at 72 °C. The final extension was performed for 10 min at 72 °C. PCR products from each DNA sample were pooled and mixed with a loading cocktail containing HD-400 size standards (Applied Biosystems). The resulting PCR products were separated in an ABI 3100 DNA Analyzer (Applied Biosystems) and genotypes were assigned with GeneMapper software (Applied Biosystems).

### Linkage analysis

Two-point linkage analyses were performed using the FASTLINK version of MLINK from the LINKAGE Program Package (provided in the public domain by the Human Genome Mapping Project Resources Centre, Cambridge, UK) [19,20]. Maximum LOD scores were calculated with ILINK from the LINKAGE Program Package. Autosomal recessive cataract was analyzed as a fully penetrant trait with an affected allele frequency of 0.001. The marker order and distances between the markers were obtained from the Marshfield database and the National Center for Biotechnology Information (NCBI) chromosome 7 sequence maps. For the initial genome scan, equal allele frequencies were assumed, while for fine mapping allele frequencies were estimated from 96 unrelated and unaffected individuals from the Punjab province of Pakistan.

### Mutation screening

Primer pairs for individual exons were designed using the primer3 program. The sequences, and annealing temperatures, and product size are shown in Table 1. Amplifications were performed in 25 μl reaction volume containing 50 ng of genomic DNA, 400 nM of each primer, 250 μM of dNTPs, 2.5mM MgCl_2_, and 0.2 U Taq DNA polymerase in the standard PCR buffer provided by the manufacturer (Applied Biosystems). PCR amplification consisted of a denaturation step at 96 °C for 5 min followed by 40 cycles, each consisting of 96 °C for 45 s followed by 57 °C for 45 s and 72 °C for 1 min. PCR products were analyzed on 2% agarose gel and purified by ethanol precipitation. The PCR primers for each exon were used for bidirectional sequencing using BigDye Terminator Ready reaction mix, according to manufacturer instructions. Sequencing products were precipitated and resuspended in 10 μl of formamide (Applied Biosystems) and denatured at 95 °C for 5 min. Sequencing was performed on an ABI PRISM 3100 Automated sequencer (Applied Biosystems). Sequencing results were assembled with ABI PRISM sequencing analysis software version 3.7 and analyzed with SeqScape software (Applied Biosystems).

**Table 1:** Primer sequences and annealing temperatures of *GJE1*.

***GJE1***	**Forward primer**	**Reverse primer**	**Product size (bp)**	**Annealing temperature (°C)**
5`UTR 1a	CATCATTCAGACCATACCACA	AACCCAGCAGAATTTACCAG	350	54
5`UTR 1b	TGTCACCATGAGGGAGATAA	GCAAATTAGTGCTGATGCTG	383	54
5`UTR 1c	TCACATCTAGCCCCATAACC	CCAAGACTCTGATTGCACCT	466	55
5`UTR 1d	TCCACTCCAGCAGTACAGAA	GTTTTGGAGCAGAGGACAAG	257	55
Exon 1a	GGTGCAATCAGAGTCTTGGT	CCTTCCCCTTTCCTGATAAT	491	54
Exon 1b	TGAGCAGAGTGAATTCGTGT	AAGAGACAGAAACCGCTGAC	474	54
Exon 1c	TTGCAGTACCACCTGTATGG	TGCCTAGAACCTGGTTACCT	452	55
Exon 2	GGTACGCACTGTGAAAAAGTT	GGTGCAAACATGGCTTTTAT	315	55

## Results

**Figure 1:**
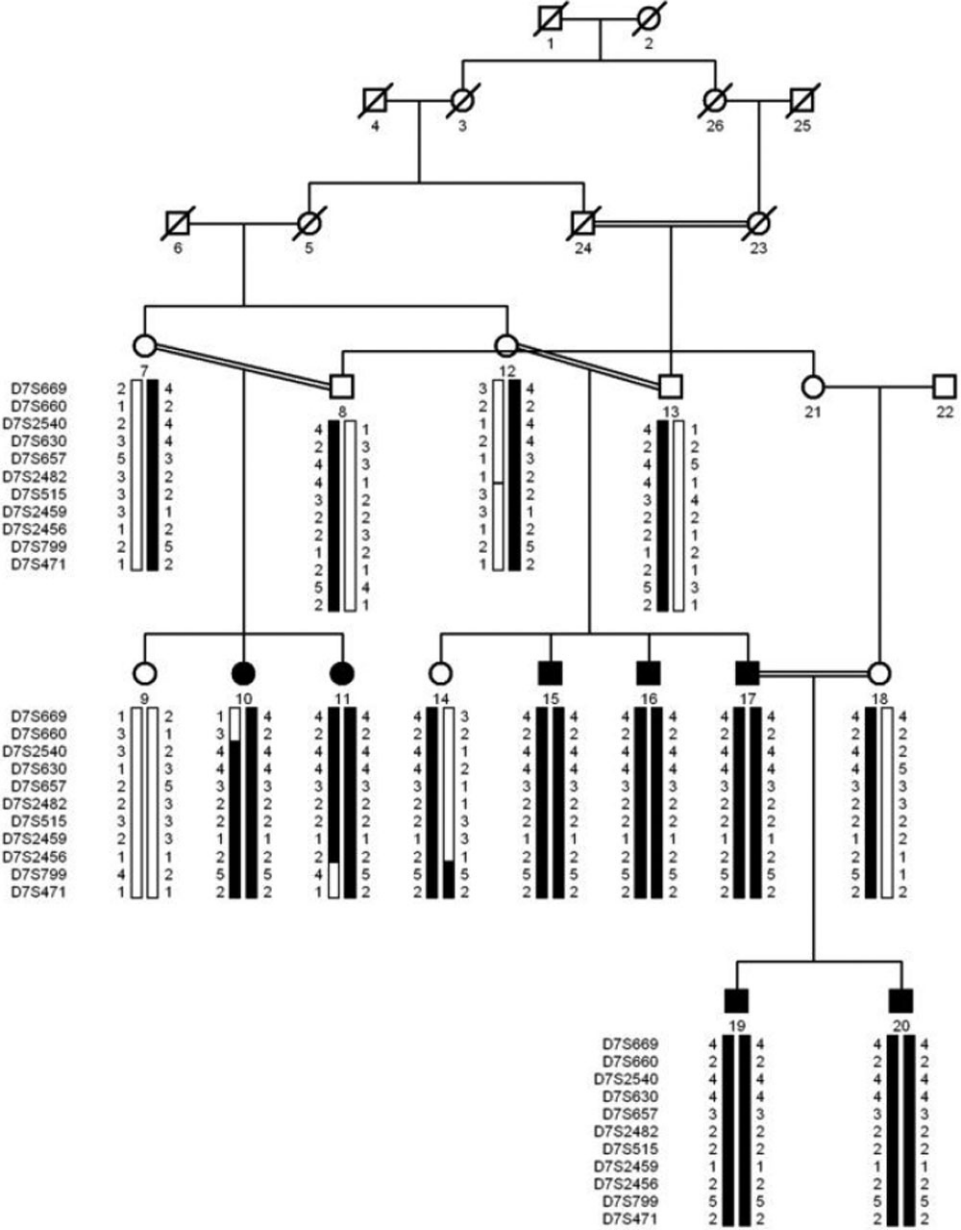
Pedigree drawing and haplotypes of chromosome 7q markers of family PKCC108. Squares are males, circles are females, and filled symbols are affected individuals; the double line between individuals indicates consanguinity and the diagonal line through a symbol is a deceased family member. The haplotypes of 11 adjacent chromosome 7q microsatellite markers are shown; alleles forming the risk haplotype  are shaded black, while alleles not co-segregating with cataract are shown in white.

**Figure 2:**
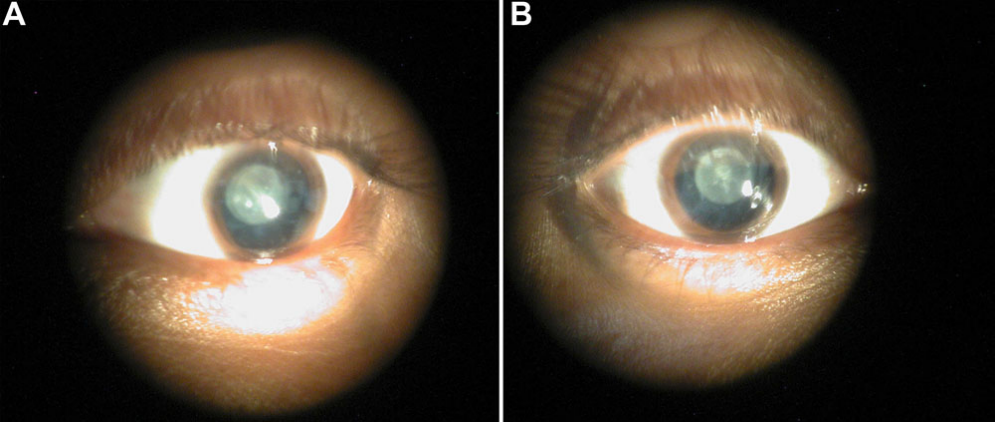
Slit lamp photographs of the affected individuals of PKCC108. **A**: Individual 16 and **B**: individual 19 of PKCC108 reveal nuclear cataracts that developed in early infancy.

The family described in this study, PKCC108 (Figure 1) is from the Punjab province of Pakistan, and a detailed medical and family history was obtained from all affected and unaffected members. According to the medical family records cataracts in all affected individuals developed in the early years of their lives. Clinical examination conducted with slit-lamp microscopy revealed nuclear cataract in both eyes of affected individuals 16 and 19 (Figure 2). No other ocular or systemic abnormalities were present in the family. Linkage to known autosomal recessive cataract loci was initially excluded by haplotype analysis using closely flanking markers (data not shown).

During the genome-wide linkage scan, LOD scores greater than 1.0 were obtained for markers D1S142, D7S630, D7S657, and D7S515. Of these, D1S142 has closely flanking markers yielding large negative LOD scores and thus was excluded from further consideration. However D7S630, D7S657, and D7S515, adjacent markers in the MD-10 mapping set, yielded LOD scores of 5.01, 4.38 and 4.17 at θ=0, respectively. Fine mapping with closely spaced markers D7S660, D7S2540, D7S2482, D7S2459, D7S2456, D7S799, and D7S471 further confirmed linkage to markers on chromosome 7q. Maximum LOD scores of 5.08, 2.94, 4.76, and 4.32 at θ=0 were obtained with markers D7S2540, D7S2482, D7S2459, and D7S2456, respectively (Table 2).

**Table 2:** Two point parametric LOD scores of chromosome 7q markers for family PKCC108.

**Marker**	**cM**	**Mb**	**0.00**	**0.01**	**0.05**	**0.09**	**0.10**	**0.20**	**0.30**	**Z_max_**	**θ_max_**
D7S669*	90.42	77.81	- ∞	2.34	2.72	2.64	2.61	2.05	1.33	2.72	0.05
D7S660	93.63	80.53	- ∞	1.04	1.51	1.52	1.51	1.18	0.71	1.53	0.07
D7S2540	97.38	83.05	5.08	4.98	4.61	4.21	4.11	3.07	1.97	5.08	0.00
D7S630*	98.44	88.38	5.01	4.92	4.54	4.16	4.06	3.04	1.96	5.01	0.00
D7S657*	104.86	92.74	4.38	4.293	3.94	3.59	3.51	2.59	1.63	4.38	0.00
D7S2482	108.59	95.06	2.94	2.88	2.65	2.42	2.37	1.76	1.14	2.94	0.00
D7S515*	112.32	101.59	4.17	4.08	3.75	3.41	3.32	2.42	1.51	4.17	0.00
D7S2459	119.81	107.33	4.76	4.67	4.29	3.91	3.81	2.79	1.74	4.76	0.00
D7S2456	120.61	107.57	4.32	4.23	3.86	3.49	3.41	2.44	1.46	4.32	0.00
D7S799	121.41	108.49	- ∞	1.28	2.32	2.46	2.46	2.11	1.44	2.46	0.10
D7S471	123.01	112.03	- ∞	0.472	1.55	1.74	1.75	1.51	0.96	1.75	0.10

The asterisk indicates markes included in genome wide scan. LOD scores were calculated at different θ-values for each marker with the FASTLINK version of MLINK from the LINKAGE program package. Maximum LOD scores for each marker were calculated using ILINK. Asterisk: STR Marker included in genome wide scan.

Visual inspection of the haplotypes further supported the linkage results of the linkage analysis. A recombination event in individual 10 established the proximal boundary of the linked region at D7S660. Similarly, there is a distal recombination event in affected individual 11 and unaffected individual 14 at D7S799. Taken together, this places the disease locus in the 27.78 cM (27.96 Mb) interval of chromosome 7, flanked by markers D7S660 and D7S799 (Figure 1). All affected individuals are homozygous for alleles of markers D7S2540, D7S630, D7S657, D7S2482, D7S515, D7S2459, and D7S2456, whereas the unaffected individuals are heterozygous carriers of the disease allele, except individual 9, who inherited the normal alleles from both parents (Figure 1).

The critical interval of 27.78 cM (27.96 Mb) is a gene-rich region and among these resides GJE1, a gap junction protein. As other gap junction proteins have been associated with autosomal recessive cataracts [7], we therefore sequenced all coding exons, exon–intron boundaries and the 5′ untranslated region of *GJE1* but did not identify any pathogenic mutations.

## Discussion

Here we report identification of a new locus for autosomal recessive cataract mapped to chromosome 7q21.11-q31.1 in a consanguineous Pakistani family (PKCC108). A maximum LOD score of 5.08 was obtained with D7S2540 at θ=0, and the disease locus co-segregates with chromosome 7q markers in 27.78 cM (27.96 Mb) interval flanked by D7S660 and D7S799. The lack of significant LOD scores other than those in the chromosome 7q region during the genome-wide linkage scan, homozygous alleles for all the affected individuals for markers of 7q, and lack of homozygosity in all the unaffected individuals strongly suggest that the disease locus maps to a new region on chromosome 7q. No previously reported cataract loci or genes are located in the critical interval.

To date, 12 loci for recessive congenital cataract have been identified, illustrating the genetic heterogeneity of the disease. Previously, nuclear cataracts segregating in consanguineous families have been mapped to chromosome 2p12, 19q13.4, and 22q12.3, in the latter of which mutations in *CRYBB3* were identified [12,16,21]. All of these families are of Pakistani origin, suggesting that nuclear cataract is prevalent in the Pakistani population.

The critical interval harbors *GJE1*, a gene that is a member of the gap junction protein family. As the lens is an avascular structure that lacks almost all cell organelles, cell–cell communication is brought about by an extensive system of gap junction proteins. Gap junction proteins GJA1 (connexin 43), GJA3 (connexin 46), and GJA8 (connexin 50) are expressed in human lens, and mutations in *GJA3* have been reported to cause cataract with different morphology [22,23]. Similarly, there are many reports of mutations in *GJA8* [24,25]. We sequenced all coding exons, exon–intron boundaries, and the 5′ untranslated region of *GJE1* but found no pathogenic mutations.

Transparency and precise shape and optical density are distinctive features of the lens that are critical for proper light refraction. Elucidating the molecular mechanisms that maintain or disrupt lens transparency is a fundamental step toward preventing cataract. Identification of new genes involved in cataract will help us understand the molecular biology of the human lens and the structural and metabolic mechanisms involved in maintenance of the transparency of the lens.
